# Exploring miRNA-Associated Signatures with Diagnostic Relevance in Glioblastoma Multiforme and Breast Cancer Patients

**DOI:** 10.3390/jcm4081612

**Published:** 2015-08-14

**Authors:** Véronique C. LeBlanc, Pier Jr Morin

**Affiliations:** Department of Chemistry and Biochemistry, Université de Moncton, 18 Antonine-Maillet avenue, Moncton, NB E1A 3E9, Canada; E-Mail: evl6741@umoncton.ca

**Keywords:** microRNAs, glioma, glioblastoma multiforme, breast cancer, cancer diagnosis, cancer therapeutics, non-coding RNAs, long non-coding RNAs

## Abstract

The growing attention that non-coding RNAs have attracted in the field of cancer research in recent years is undeniable. Whether investigated as prospective therapeutic targets or prognostic indicators or diagnostic biomarkers, the clinical relevance of these molecules is starting to emerge. In addition, identification of non-coding RNAs in a plethora of body fluids has further positioned these molecules as attractive non-invasive biomarkers. This review will first provide an overview of the synthetic cascade that leads to the production of the small non-coding RNAs microRNAs (miRNAs) and presents their strengths as biomarkers of disease. Our interest will next be directed at exploring the diagnostic utility of miRNAs in two types of cancer: the brain tumor glioblastoma multiforme (GBM) and breast cancer. Finally, we will discuss additional clinical implications associated with miRNA detection as well as introduce other non-coding RNAs that have generated recent interest in the cancer research community.

## 1. Introduction

Tremendous effort has been dedicated in recent years to elucidating the underlying functions of non-coding RNAs, including the small microRNAs (miRNAs), in numerous types of cancer. Several studies have characterized the roles played by miRNAs in primary tumors and have positioned these molecules as significant drivers of malignancy [1,2,3]. Importantly, such work has put the light on miRNAs as appealing cancer biomarkers, notably due to their significant stability and their ability to reveal crucial information on tumor grade and treatment response [4,5,6]. With an emphasis on *in vivo* human studies, this review first presents the potential advantages associated with miRNAs as cancer biomarkers and subsequently discusses studies that have identified miRNAs with diagnostic relevance in two types of cancers: glioblastoma multiforme and breast carcinomas. Finally, we introduce examples of work that have assessed the usefulness of miRNAs in other, non-diagnostic, clinical applications as well as present additional non-coding RNAs with diagnostic relevance to cancer.

## 2. MiRNAs: An Overview

MiRNA biogenesis usually starts with the transcription of miRNA genes by RNA polymerase II to generate a primary miRNA transcript termed pri-miRNA [7,8]. This capped and polyadenylated structure is further processed in the nucleus by the microprocessor complex comprised of the RNase III enzyme Drosha and the cofactor DiGeorge syndrome critical region gene 8 (DGCR8) to generate a pre-miRNA that is subsequently exported to the cytoplasm via Exportin-5 [7,9,10,11,12]. The RNase III enzyme Dicer performs pre-miRNA cleavage to yield a 20–24 nucleotide duplex miRNA from which the mature miRNA sequence will associate with Argonaute and other proteins to form the miRNA-induced silencing complex (miRISC) [13,14]. MiRISC can interact, via imperfect base pairing, with the 3′-untranslated region (3′-UTR) of transcript targets and alter their expression via translational repression or mRNA destabilization. Complementarity between the seed region of the miRNAs (nucleotides 2–8) and nucleotides of the target mRNA plays a pivotal role in target recognition and silencing [15]. Recent evidences suggest that miRNA/transcript target interaction can also occur in the 5′-UTR or within the coding region of some mRNAs [16,17].

There are multiple arguments that support the investigation of miRNAs as biomarkers for diseases. MiRNAs can notably be packaged into exosomes, small bioactive reservoirs secreted by cells, and subsequently regulate transcript targets of recipient cells [18]. Previous work has demonstrated that miRNAs secreted by cancer cells can have various effects such as increased drug resistance and transformation of target cells [19,20]. Isolation and characterization of the molecules present in exosomes for diagnostic and prognostic purposes have been performed in different types of cancer including gliomas and breast cancer [21,22], the focus of the current review. Accordingly, miRNAs are thus present in various body fluids including serum, urine and saliva, making them collectable and quantifiable via non-invasive methods [23,24,25]. Furthermore, miRNAs are significantly stable in a variety of biological specimens such as blood, urine and postmortem formalin-fixed paraffin-embedded (FFPE) tissues [26,27,28]. MiRNA isolation from these sources is thoroughly documented and their subsequent quantification can be performed with a variety of techniques such as quantitative reverse transcription polymerase chain reaction (qRT-PCR), miRNA microarrays or next-generation sequencing to name a few [29,30,31]. Finally, miRNA levels, in primary tissues and in circulating samples, have also been associated with different clinical parameters in cancer such as metastatic progression and response to chemotherapeutic agents [32,33]. MiRNAs thus possess a number of criteria that position them as appealing cancer biomarkers, and the subsequent sections will focus on the diagnostic potential of miRNAs in two types of cancer.

## 3. Glioblastoma Multiforme and MiRNAs

Glioblastoma multiforme (GBM) is the most aggressive and frequently diagnosed primary brain tumor [34]. This grade IV glioma is highly malignant and the prognosis for patients diagnosed with a GBM remains poor with a median survival rate between 12 to 15 months [35,36]. Standard of care consists of surgical resection of the tumor followed by a combination of radiotherapy and chemotherapy [37]. At the molecular level, GBMs can be divided into four subtypes based on the following gene signatures: classical, mesenchymal, neural and proneural [38]. Amplification of the epidermal growth factor receptor (*EGFR*) gene is a frequent occurrence in primary GBMs as well as mutations of phosphatase and tensin homolog (*PTEN*) tumor suppressor gene [39,40]. Interestingly, selected biomarker status is progressively being considered in the clinical assessment and management of certain subtypes of brain tumors such as the evaluation of O6-methylguanine-DNA methyltransferase (*MGMT*) promoter methylation status in elderly patients diagnosed with a GBM [41].

MiRNAs are appealing therapeutic targets and potential biomarkers of GBMs [42]. Deregulation of these molecules, capable of impacting several processes including cell proliferation, cell cycle regulation and angiogenesis, underlie GBM pathogenesis [43]. Not surprisingly, numerous miRNAs are differentially expressed in primary GBM tumors with targets that notably include transcript coding for proteins with oncogenic or tumor suppressive functions. Early work that assessed miRNA expression via microarray in tissue samples obtained from nine primary GBM patients and ten GBM cell lines notably revealed elevated miR-221 levels in this tumor [44]. It was subsequently demonstrated that the tumor suppressor p27(Kip1), which displays reduced protein levels in GBMs, was a direct miR-221 target [45]. Two additional tumor suppressors, CDKN1A (p21) and CDKN2A (p16), were shown to be direct targets of miR-10b, a miRNA significantly upregulated in malignant gliomas [46]. MiR-21 and miR-26a are also overexpressed in primary GBM tumors and can alter PTEN expression [47,48]. MiR-21 has been associated with GBM cell proliferation and response to cisplatin by targeting FOXO1 [49]. MiR-21 can also impact GBM cell proliferation by regulating Fas ligand (FASLG) protein expression [50]. Interestingly, miR-21 downregulation significantly reduces the oncogenic potential of GBM cell lines independently of PTEN status and affects Akt activity as well as EGFR levels [48]. Expression of the latter is also regulated, directly or indirectly, in GBMs by miRNAs such as miR-7, miR-34a, miR-146b-5p and miR-219-5p [51,52,53,54]. The strong invasiveness observed in GBMs is also mediated by differential expression of miRNAs including miR-218, a miRNA that directly targets LEF1 and affects MMP-9 protein levels [55], as well as miR-491-5p and miR-491-3p, which notably target CDK6 and other molecular players linked with GBM cell invasion [56].

While examples abound of modulated miRNAs in primary GBM tumors, miRNAs are also released by GBMs and can be subsequently isolated and quantified in various body fluid samples, thus positioning these molecules as circulating biomarkers of malignancy. A study revealed significant miR-128 upregulation and miR-342-3p downregulation in blood samples of GBM patients when compared with healthy individuals [57]. Subsequent work confirmed altered miR-128 and miR-342-3p levels in plasma samples of GBM patients and showed that these miRNAs positively correlated with histopathological grades of glioma [58]. It is important to mention that miR-128 levels, as opposed to circulating samples, are reduced in primary GBM specimens which positions this miRNA as an interesting therapeutic target for this malignancy [59,60]. Monitoring miRNAs in pre-operative plasma samples also revealed increased miR-21 levels in GBMs [61]. MiR-21 was also identified as significantly upregulated in extra-cellular vesicles (EVs) isolated from cerebrospinal fluid (CSF) of GBM patients when compared with EVs from healthy subjects further supporting the diagnostic relevance of miR-21 [62]. A similar study investigated the miRNA content of serum microvesicles collected from 25 GBM patients and notably highlighted a correlation between miR-320 and miR-574-3p levels and GBM diagnosis [63]. Overall, these studies provide a glimpse of the potential associated with miRNAs as non-invasive biomarkers for GBM diagnosis.

## 4. Breast Cancer and MiRNAs

Breast cancer, unlike GBM, is at the opposite end of the cancer incidence being the most frequent carcinoma observed in women in the United States. It is also the cancer that ranks second on the list of estimated deaths per cancer types for the same gender [64]. As for other types of cancer, early breast cancer detection is of crucial importance to improve the chance of patient survival. Substantial profiling of primary breast tumors has highlighted a variety of subtypes, such as luminal A, luminal B, HER2-enriched and basal-like, with different molecular background and clinical outcomes [65]. The latter subtype also includes triple-negative breast cancer, which lacks immunohistochemical detection of estrogen receptor (ER), progesterone receptor (PR) and human epithelial growth factor receptor-2 (HER-2) [66]. Mutations of the *BRCA1* gene, besides conferring a significant lifetime risk of breast cancer diagnosis [67], are also frequently observed in the triple-negative phenotype [68].

**Figure 1:**
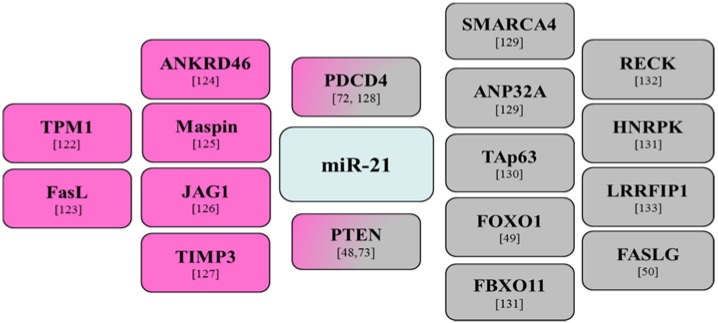
MiR-21 validated targets in glioblastoma multiforme and breast cancer studies. Targets in breast cancer are shown in pink and targets in glioblastoma multiforme are shown in gray.

Pioneering work performed in tumor samples collected from a cohort of 344 patients diagnosed with primary breast cancer revealed strong miR-21 expression [69]. MiR-21 was correlated with limited disease-free survival in early stage patients. Subsequent work further positioned miR-21 as an important miRNA underlying breast cancer as it displayed strong expression in triple-negative primary breast cancers as well as in breast cancer patients with short disease-free survival [70,71]. Interestingly, and as previously observed in GBMs, miR-21 can target the tumor suppressor protein programmed cell death 4 (PDCD4) in human breast cancer cells [72]. This miRNA can also target, as in GBMs, PTEN in breast cancer and impact the response to chemotherapeutic agents [73]. An overview of the principal miR-21 validated targets in GBMs and breast cancer is presented in Figure 1.

**Figure 2:**
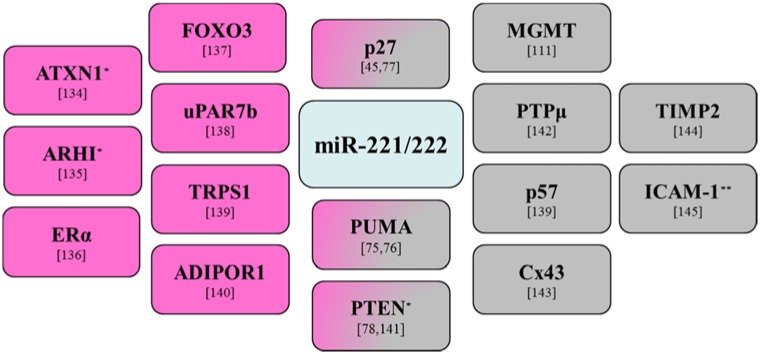
MiR-221/222 validated targets in glioblastoma multiforme and breast cancer studies. Targets in breast cancer are shown in pink and targets in glioblastoma multiforme are shown in gray. * Targets regulated by miR-221 alone. ** Target regulated by miR-222 alone.

The former study also demonstrated elevated miR-221 and miR-222 expression in the triple-negative specimens. MiR-221/222 is upregulated in HER2-positive primary human breast cancer tissues and has been linked with tamoxifen resistance [74]. MiR-221/222 deregulation leads to modulation of p53 upregulated modulator of apoptosis (PUMA), a pro-apoptotic protein, in human gliomas and breast cancer cells [75,76]. Interestingly, miR-221 can regulate the expression of the tumor suppressor proteins p27 and PTEN in GBMs and breast cancer models [45,77,78]. An overview of the principal miR-221/222 validated targets in GBMs and breast cancer is shown in Figure 2.

MiR-155 is also one of the first miRNAs to be reported as significantly deregulated in primary breast tumors [79]. Several subsequent studies confirmed miR-155 overexpression in breast cancer tissues [80,81,82] and recent work presented the tumor protein p53-induced nuclear protein 1 (TP53INP1) as a miR-155 target in MCF-7 cells [83]. MiR-10b is another example of a miRNA with oncogenic properties that is differentially expressed in primary breast cancer. MiR-10b levels in primary breast carcinomas correlate with several clinical parameters including tumor size, pathological grading, clinical staging and lymph node metastasis [84,85]. While these oncogenic miRNAs are only the tip of the iceberg when it comes to deregulated miRNAs in breast cancer, it is important to mention that several deregulated miRNAs with tumor suppressive functions have also been identified. Examples include miR-125b, a miRNA that directly targets the ETS1 proto-oncogene in breast cancer [86], which exhibits differential expression between primary and metastatic breast tumors [87] and was most recently reported to impact breast cancer chemoresistance in blood serum samples of breast cancer patients [88]. Downregulation of miR-205, a direct regulator of HER3 receptor expression in breast cancer [89], was observed in primary tumor tissues *versus* adjacent benign breast tissue [90] and subsequent work in FFPE tissues of patients with early breast cancer further demonstrated that differential expression of this miRNA could impact overall survival [91]. MiR-206 levels were measured in cancer tissues of 128 breast cancer patients via qRT-PCR and revealed reduced expression when compared with normal adjacent tissues [92]. The tumor suppressive properties of miR-206 are likely explained via modulation of its validated target Cyclin D1 [93]. Interestingly, Cyclin D1 is a well-characterized occurrence in primary breast cancer [94] and this further highlights the potential importance of the miR-206-Cyclin D1 axis in this malignancy.

As for GBMs, miRNAs have also been identified in circulating samples of breast cancer patients and have been investigated further for their diagnostic potential [95]. Early work revealed elevated miR-195 levels in blood samples collected from pre-operative breast cancer patients when compared with samples processed from matched controls [96]. The same study also revealed circulating miR-155 overexpression in multiple types of cancer. MiR-155 serum levels were subsequently reported to identify healthy subjects from breast cancer patients further strengthening its diagnostic potential [97,98]. As in primary breast cancer tissues, differential expression of miR-21 in circulating samples has been demonstrated in numerous studies. MiR-21 levels measured by qRT-PCR in serum samples collected from 102 breast cancer patients and 20 healthy female donors highlighted the capacity of this miRNA to discriminate between the two groups [99]. Subsequent work in different cohorts of breast cancer patients further reported miR-21 differential expression between circulating samples collected from patients and samples obtained from healthy individuals [100,101]. Novel studies have revealed signatures of multiple miRNAs associated with breast cancer [102,103] and validation of such footprints in other cohorts of breast cancer patients is foreseen to better decipher their clinical relevance.

## 5. MiRNAs as Biomarkers: Beyond Diagnostic

Several miRNAs with diagnostic potential in GBMs and in breast cancer have been presented up to this point and a list of commonly deregulated miRNAs in these two types of cancer is presented in Table 1.

In addition and as alluded in this article, the clinical usefulness of miRNAs reach beyond their capabilities of diagnosing malignancy. Indeed, miRNAs have also been investigated as prognostic markers. Specific examples in brain tumors include miR-328 which is strongly expressed in glioma cells *in vivo* and is associated with poor overall patient survival [104] as well as elevated miR-210 levels in serum samples of GBM patients which correlate with poor survival [105]. In breast cancer, miRNA expression by qRT-PCR was performed in blood samples collected from patients and healthy individuals and revealed that miR-200c and miR-141 levels correlated with overall survival [106]. A signature comprising of miR-18b, miR-103, miR-107 and miR-652 efficiently predicted overall survival in serum samples obtained from a cohort of 60 triple-negative breast cancer patients [107]. Examples of miRNAs as potential biomarkers of therapeutic response also exist. In GBMs, elevated MGMT levels confer resistance to the alkylating agent temozolomide (TMZ) [108]. MiR-181d was shown to act as a suitable predictor of TMZ response in GBM cases and to directly regulate MGMT expression [109]. Other examples of miRNAs capable of modulating MGMT expression include miR-221, miR-222, miR-603, miR-648 and miR-767-3p, further supporting the underlying importance of these non-coding RNAs in TMZ response in GBMs [110,111,112]. MiRNAs such as let-7i, miR-93, miR-130a, miR151-3p, miR-423-5p, miR-938, miR-1238, and miR-1280 have also been correlated with TMZ response in GBMs independently of MGMT status [113,114]. In breast cancer, elevated miR-125b levels were detected in blood serum samples collected from 56 patients and were associated with poor chemotherapeutic response [86]. A study in plasma samples of breast cancer patients also linked circulating miR-210 levels with trastuzumab resistance [115]. While this review has focused on the diagnostic potential of miRNAs, there is clear evidence that these molecules also possess additional clinical properties.

## 6. Conclusions

In addition to miRNAs, it is important to mention that other non-coding RNAs such as long non-coding RNAs (lncRNAs) are appealing molecules to investigate for their diagnostic potential in different types of cancer. While the information available regarding lncRNAs as potential cancer biomarkers in human *in vivo* models is not as vast as for the miRNAs, interesting work is starting to emerge in this research area. Two studies notably reported elevated HOX antisense intergenic RNA (HOTAIR) lncRNA levels in blood samples collected from cervical and colorectal cancer patients and correlated this observation with poor prognosis [116,117]. In gliomas, the identification of subtypes based on lncRNA expression provided pioneering work for the clinical relevance of lncRNAs in brain tumors [118]. MEG3, an lncRNA with tumor-suppressive functions, displayed significant downregulation in glioma tissue samples when compared with adjacent normal tissues and its overexpression in two GBM cell lines promoted apoptosis [119]. Early work in breast cancer FFPE tissues notably showed that strong HOTAIR expression was linked with ER and PR expression [120] and a recent study observed elevated lncRNA RP11-445H22.4 levels in serum samples collected from a cohort of 136 breast cancer patients [121].

In conclusion, whether to monitor treatment response in GBMs or for early breast cancer detection, several examples exist that illustrate non-coding RNAs with diagnostic, prognostic and therapeutic response assessment potential. Deciphering the circulating miRNA footprint associated with these malignancies is undoubtedly of great clinical interest and tremendous progress has been made in this research area in recent years. Nevertheless, challenges remain before non-coding RNAs are leveraged as bona fide biomarkers in the two types of cancer explored in this review and further investigation is needed in this research field to unveil clinically relevant miRNA-based signatures.

**Table 1:** Commonly deregulated microRNAs (miRNAs) in primary and circulating glioblastoma multiforme (GBM) and breast cancer (BC) samples. BC: Differential expression of miRNA only reported in breast cancer.

miRNA	Differential expression	Sample type	References
miR-7-5p	Downregulated	Primary tumors	[146,147]
miR-10b	Upregulated	Primary tumors Serum (BC)	[148,149,150,151]
miR-17/92	Upregulated	Primary tumors	[152,153]
miR-21	Upregulated	Primary tumors Plasma	[61,69,154,155]
miR-155	Upregulated	Primary tumors Serum (BC)	[79,150,156]
miR-182	Upregulated	Primary tumors	[157,158]
miR-221	Upregulated	Primary tumors	[44,70]
miR-222	Upregulated	Primary tumors	[44,74]
